# Perceptions of Facilitators and Barriers to Implementation of Falls Prevention Programs in Primary Health Care Settings in China

**DOI:** 10.1001/jamanetworkopen.2022.28960

**Published:** 2022-08-26

**Authors:** Pengpeng Ye, Ye Jin, Yuliang Er, Xuejun Yin, Yao Yao, Bingqin Li, Jing Zhang, Rebecca Ivers, Lisa Keay, Leilei Duan, Maoyi Tian

**Affiliations:** 1 The George Institute for Global Health, Faculty of Medicine and Health, UNSW Sydney, Sydney, Australia; 2 National Centre for Non-Communicable Disease Control and Prevention, Chinese Centre for Disease Control and Prevention, Beijing, China; 3 China Centre for Health Development Studies, Peking University, Beijing, China; 4 Social Policy Research Centre, UNSW Sydney, Sydney, Australia; 5 School of Population Health, Faculty of Medicine and Health, UNSW Sydney, Sydney, Australia; 6 School of Optometry and Vision Science, Faculty of Medicine and Health, UNSW Sydney, Sydney, Australia; 7 School of Public Health, Harbin Medical University, Harbin, China

## Abstract

**Importance:**

Falls have become a major public health issue in China with population aging. Although falls prevention for older community-dwelling people has been included in the National Essential Public Health Service Package since 2009, there is limited understanding of the implementation of this program.

**Objective:**

To identify the associated factors and provide recommendations to inform the better implementation of falls prevention in the Chinese primary health care system.

**Design, Setting, and Participants:**

This qualitative study was conducted in 3 purposively selected cities in China from March 1 to June 7, 2021. Health administrators from the local health commission or bureau, staff members from local Centers for Disease Control and Prevention and primary health care facilities and community-dwelling older people were recruited, using a combination of purposive sampling and snowball sampling.

**Main Outcomes and Measures:**

In-depth interviews were conducted with health administrators and focus groups with other participants. Data analysis followed the guidance of the Consolidated Framework for Implementation Research. Study outcomes included facilitators and barriers of implementing falls prevention for older people in the Chinese primary health care settings. A framework with recommendations was developed to inform the future intervention implementation.

**Results:**

Among a total of 130 participants interviewed, 77 (59.2%) were female and the mean (SD) age was 47.4 (16.7) years. Clear recognition of the challenges and benefits of falls prevention, adaptive regionally tailored guidance plans, and continuous governmental policy and financial support were the major facilitators, whereas the major barriers consisted of insufficient confidence in delivering interventions and poor understanding of the falls burden, low recognition of the importance of falls prevention, limited multisectoral collaboration, and weak financial incentives. A 7-strategy embedded framework—including data-driven surveillance, audit and feedback, implementation strategy, workforce strengthening, community empowerment, internal services integration, and external enabling environment—was developed to foster successful implementation.

**Conclusions and Relevance:**

This qualitative study identified major facilitators and barriers to the implementation of falls prevention for older people at the primary care level, which have the potential to contribute to better implementation of falls prevention for older people in the Chinese primary health care system.

## Key Points

**Question:**

What are the facilitators and barriers of implementing falls prevention for older people in Chinese primary health care (PHC) settings?

**Findings:**

This qualitative study of 130 participants identified 14 facilitators and 20 barriers in implementing falls prevention for older people in PHC settings. A recommendation framework was then developed to respond to those barriers.

**Meaning:**

This study’s findings suggest the challenges of implementing falls prevention for older people in Chinese PHC settings and also provide a high-level framework to inform future better implementation.

## Teaser

This qualitative study identifies facilitators and barriers to implementation of falls prevention programs in the Chinese primary health care system and provides recommendations.

## Introduction

Falls have been recognized as a complex but preventable health issue among older people.^1^ With rapid population aging in China, falls have become a major public health issue.^2,3^ In 2019, among Chinese people aged 60 years and above, the incidence rate of falls was 3799.4 per 100 000 population, and the mortality rate was 39.2 per 100 000 population.^3^ Diverse falls prevention programs have been implemented, targeting different areas, organizations, and populations.^4^ However, most previous programs were either small in scale or implemented in the context of research without long-term sustainability.^4^

In 2009, the Chinese Central Government launched the National Essential Public Health Service Package (NEPHSP), with the primary aim to strengthen the primary health care (PHC) system.^5^ In the NEPHSP, PHC clinicians, regardless of urban or rural areas, were required to provide annual complimentary health management service for community residents aged 65 years and older, including falls prevention.^6^ Despite the NEPHSP having been implemented for more than a decade, there have been no investigations into the barriers and facilitators of implementing falls prevention for older people. This study, therefore, aims to identify the associated factors, and further provide strategies for future better implementation of falls prevention.

## Methods

### Study Design and Sites

**Figure 1.**
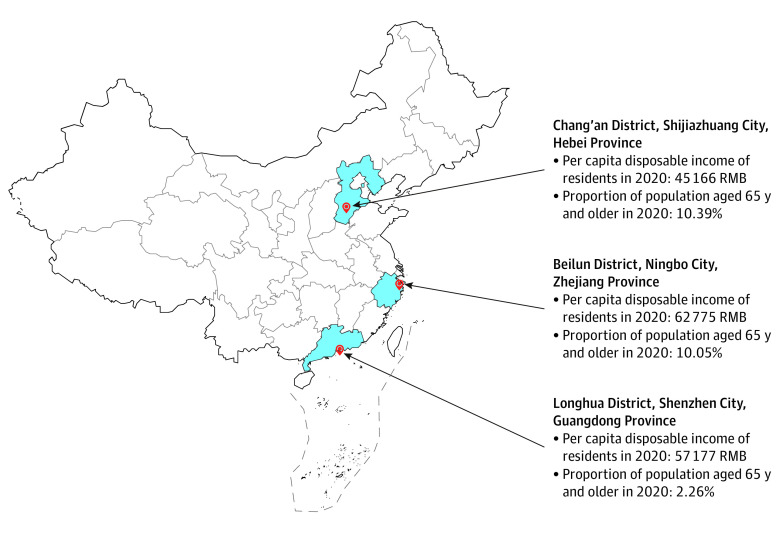
Geographic Location, Per Capita Disposable Income of Residents, and Proportion Of Population Aged 65 Years and Older of the 3 Study Sites In 2020 To convert RMB to US dollars, divide by 6.67 as of August 2, 2022.

This qualitative study was conducted in 3 purposively selected study sites with diverse geographic locations and socioeconomic status^7,8,9,10,11,12^: Chang’an District of Shijiazhuang City from Hebei Province, Beilun District of Ningbo City from Zhejiang Province, and Longhua District of Shenzhen City from Guangdong Province (Figure 1; eMethods in the Supplement). The Consolidated Criteria for Reporting Qualitative Research (COREQ) reporting guideline was used to guide the reporting of this study (eMethods in the Supplement).^13^ This study was approved by the Human Research Ethics Committee, University of New South Wales and the Ethical Review Committee of National Center for Non-Communicable Disease Control and Prevention, Chinese Center for Disease Control and Prevention.

### Study Participants and Sampling

Study participants consisted of fall-prevention service professionals and consumers. Service professionals included health administrators from the local health commission/bureau and staff members from local Center for Disease Control and Prevention (CDC) and PHC institutions. They were eligible if they had worked more than 3 months in their organizations, were responsible for or providing technical support for falls prevention in the NEPHSP and willing to sign informed consent. Older residents aged at least 65 years as consumers were eligible if they lived in the community under the jurisdiction of selected PHC institutions and were willing to provide informed consent, whereas those with serious medical conditions not suitable for interviews were excluded. Written informed consent, including permission to be audio recorded, was obtained before commencing the interviews and discussions. A combination of purposive sampling and snowball sampling was adopted to recruit participants (eMethods in the Supplement).

### Data Collection

In-depth interviews were conducted with health administrators, mainly because of scheduling difficulty and ensuring privacy so that participants were able to share views freely, by 1 interviewer (P.Y.) and 2 notetakers (Y.E. and Y.J.). Focus group discussions were used for the other 3 stakeholder groups by 1 moderator (Y.E.) and 2 notetakers (P.Y. and Y.J.). The interview and discussion guides were developed based on the Consolidated Framework for Implementation Research (CFIR) (eMethods in the Supplement).^14,15^ All guides were pilot tested and refined prior to the formal interview and discussion. All interviews and discussions were conducted face-to-face in Mandarin Chinese. Key information was confirmed with the participants to avoid possible misunderstandings. The number of in-depth interviews and focus group discussions were determined by a priori thematic saturation criteria.^16^ The number of participants (6 to 8 people per focus group discussion) is consistent with previous recommendations.^17,18^

### Data Analysis

All interviews and discussions were audio recorded and transcribed verbatim. Inductive and deductive approaches were combined to analyze the data. First, 2 researchers (P.Y. and Y.J.) independently reviewed the transcripts and inductively generated preliminary codes about influencing factors around emergent concepts. Second, similar preliminary codes were grouped into themes and then deductively mapped to the CFIR. A coding framework was generated using constant comparison to establish a hierarchy of conceptual codes until no new themes were identified. The coding discrepancies were discussed and resolved by all research team members to optimize intercoder reliability. For the strategy design, suggestions from interviews were inductively summarized, linked to the implementation strategies compiled by the Expert Recommendations for Implementation Change (ERIC) project using the CFIR-ERIC matching tool,^19^ and then iteratively adapted and refined through discussion within the research team and consultation from the external experts panel (eMethods in the Supplement). Finally, each barrier was further grouped according to potentially shared strategies and summarized into a recommendation framework. All quotations presented in this study were translated into English through forward-translation and back-translation processes to ensure rigor. Each cited quotation was marked by participant role and their study number to avoid identifiable information. Data coding and analysis were conducted in Chinese using NVivo, version 12 (QSR International) from May to October 2021.

## Results

A total of 130 participants (mean [SD] age, 47.7 [16.7] years; 77 female participants [59.2%]) were invited for either in-depth interviews (6 interviews with 50 to 70 minutes for each) or focus group discussions (19 discussions with about 6 participants in each session lasting for 90 minutes). About half of the service professionals have worked more than 10 years in their positions, with 12 service professionals working in age-specific areas for more than 10 years (Table 1). Facilitators and barriers are listed in Table 2 (eTable 1 and 2 in the Supplement).

**Table 1.** Demographic Characteristics of Participants in 3 Study Sites

Demographic characteristic	No. (%)				
	In-depth interview	Focus group discussion			Total (N = 130)
	Health administrators (n = 6)	Technical supporters (n = 38)	Primary health care clinicians (n = 45)	Community-dwelling older people (n = 41)	
Gender					
Male	6 (100)	12 (31.6)	16 (35.6)	19 (46.3)	53 (40.8)
Female	0	26 (68.4)	29 (64.4)	22 (53.7)	77 (59.2)
Age, mean (SD), y	50.0 (3.1)	35.7 (5.6)	36.7 (7.0)	70.4 (3.4)	47.7 (16.7)
Education					
College and above	6 (100)	38 (100)	45 (100)	5 (12.2)	94 (72.3)
High school and middle school	0	0	0	26 (63.4)	26 (20.0)
Primary school or below	0	0	0	10 (24.4)	10 (7.7)
Years of working					
5 y or below	0	5 (13.2)	6 (13.3)	NA	11 (12.4)
5 to 10 y	0	15 (39.5)	19 (42.2)		34 (38.2)
10 y and above	6 (100)	18 (47.4)	20 (44.4)		44 (49.4)
Years of aging-related work					
5 y or below	0	5 (13.2)	8 (17.8)	NA	13 (14.6)
5 to 10 y	3 (50.0)	30 (78.9)	31 (68.9)		64 (71.9)
10 y and above	3 (50.0)	3 (7.9)	6 (13.3)		12 (13.5)

Abbreviation: NA, not applicable.

**Table 2.** The Facilitators and Barriers to Implementing Falls Prevention for Older People in Chinese PHC Settings Spanned Across 5 Domains of the CFIR

CFIR domains	Facilitators		Barriers	
	Service professionals	Service consumers	Service professionals	Service consumers
Intervention characteristics	Governmental policy and financial support Region-tailored guidance plan Recognition of major challenges	NA	Lack of confidence in the evidence strength and quality No performance assessment indicators Poor integration within the NEPHSP No dedicated budget	NA
Outer setting	Good awareness of increasing utilization of fall-prevention service Scale-up of falls prevention required in national policies	Good awareness of falls prevention based on previous experience	Poor understanding of specific fall-prevention needs Perceiving falls not as an independent health issue Limited collaboration with other organizations An absence of a national action plan or guideline No incentives from outside	Limited knowledge with a fatalistic view Poor accessibility of easy-to-understand health information Low acceptability of the fall-prevention intervention
Inner setting	Culture in valuing PHC Mature structural characteristics, secure networks and reliable communications Recognition of fall-prevention value	NA	Lack of data support Falls prevention not prioritized within the NEPHSP Low financial incentives within PHC institutions Lack of training and capacity building resources	NA
Characteristics of individuals	Consistency of individual positive attitudes and their organization’s mission and values	NA	Insufficient professional knowledge and skills Low confidence among inexperienced PHC staff	NA
Process	Guidance plan well-developed before the implementation Experienced senior staff or managers led the implementation Positive influence from experienced senior staff or managers Positive influence from community opinion leaders	NA	Few opportunities to engage other organizations Limited audit and feedback	NA

Abbreviations: CFIR, Consolidated Framework for Implementation Research; PHC, primary health care; NA, not applicable; NEPHSP, National Essential Public Health Service Package.

### Intervention Characteristics

As a government-funded service package, NEPHSP has a well-established region-tailored guidance plan refined on an annual basis. Service professionals reported 3 major challenges to fall-prevention implementation: (1) the large number of older residents to be served, (2) insufficient PHC clinicians, and (3) the low acceptability of interventions among older people. “Six PHC providers needed to serve more than 3000 older residents. Some older people complained that fall-prevention services cost their grandchild-care time without benefit” (PHC clinician 0104).

Specific barriers are listed in Table 2. The current interventions were limited to health promotion (eg, distributing educational materials, organizing expert lectures, and broadcasting videos). CDC staff and PHC clinicians felt hesitant to provide these interventions due to the low confidence in the evidence strength and quality. “No one could ensure the effectiveness of education measures, but we were still required to organize these activities for older residents” (PHC clinician 0102).

In addition, there were no performance assessment indicators and dedicated budget for falls prevention in the NEPHSP. The integration within the same service item or with other service items (eg, hypertension management or diabetes management) was limited.

### Outer Setting

Despite that fall-related awareness, experience, and policies could facilitate the implementation (Table 2), service professionals acknowledged that they did not well understand the specific needs of local older residents. Most older people lacked essential knowledge about fall-related risks and took falls as an inevitable part of life as they age. Older people who were illiterate had poor accessibility to easy-to-understand fall-prevention information. In addition, many older people hesitated to accept further interventions due to the poor health gains from the current service. “People would fall when they were old. It could not be prevented” (Older person 0104).

The service provider organizations had limited collaboration with other government departments, civil societies, private sectors, and academic institutions. Service professionals highlighted the urgent need for a national action plan or guideline to provide specific evidence-based fall-prevention guidance in PHC settings. There were no outside financial incentives for service professionals.

### Inner Setting

Despite service professionals recognizing the value of falls prevention to improve older people’s health (Table 2), they did not prioritize falls prevention in the allocation of the workforce and budget in the NEPHSP. They argued that the main reason was the lack of data to determine the local burden of falls among older residents. “I knew some older people fell and got injured, but they were accidental cases” (Health administrator 0102).

Most PHC clinicians cited the low financial incentives within their organizations as a barrier. PHC clinicians with less experience emphasized that they needed more capacity-building resources to acquire professional fall-prevention concepts and skills.

### Characteristics of Individuals

Most service clinicians’ positive attitudes toward falls prevention were consistent with their organization’s mission and values (Table 2). However, very few CDC staff and PHC clinicians thought they had sufficient fall-prevention knowledge and skills to serve the older people. Inexperienced PHC clinicians showed low confidence in implementing fall-prevention interventions. “Sometimes, I did not know how to accurately reply to older people's questions about falls prevention” (PHC clinician 0206).

### Process

Three elements of the process were identified as facilitators. For example, the region-tailored guidance plan was well-developed 1 to 3 months prior to the new round of implementation (Table 2). However, external organizations were rarely engaged to facilitate fall-prevention decisions and implementation in PHC settings. “Local health administrators, CDC technicians and PHC providers were involved in the development of the annual regional guidance plan” (CDC staff 0103).

Despite personal and team debriefing being regularly organized among PHC clinicians to share the implementation progress, very limited feedback was provided to them to understand the quality of current interventions and the older people’s attitudes toward these interventions.

### Strategy Design

**Figure 2.**
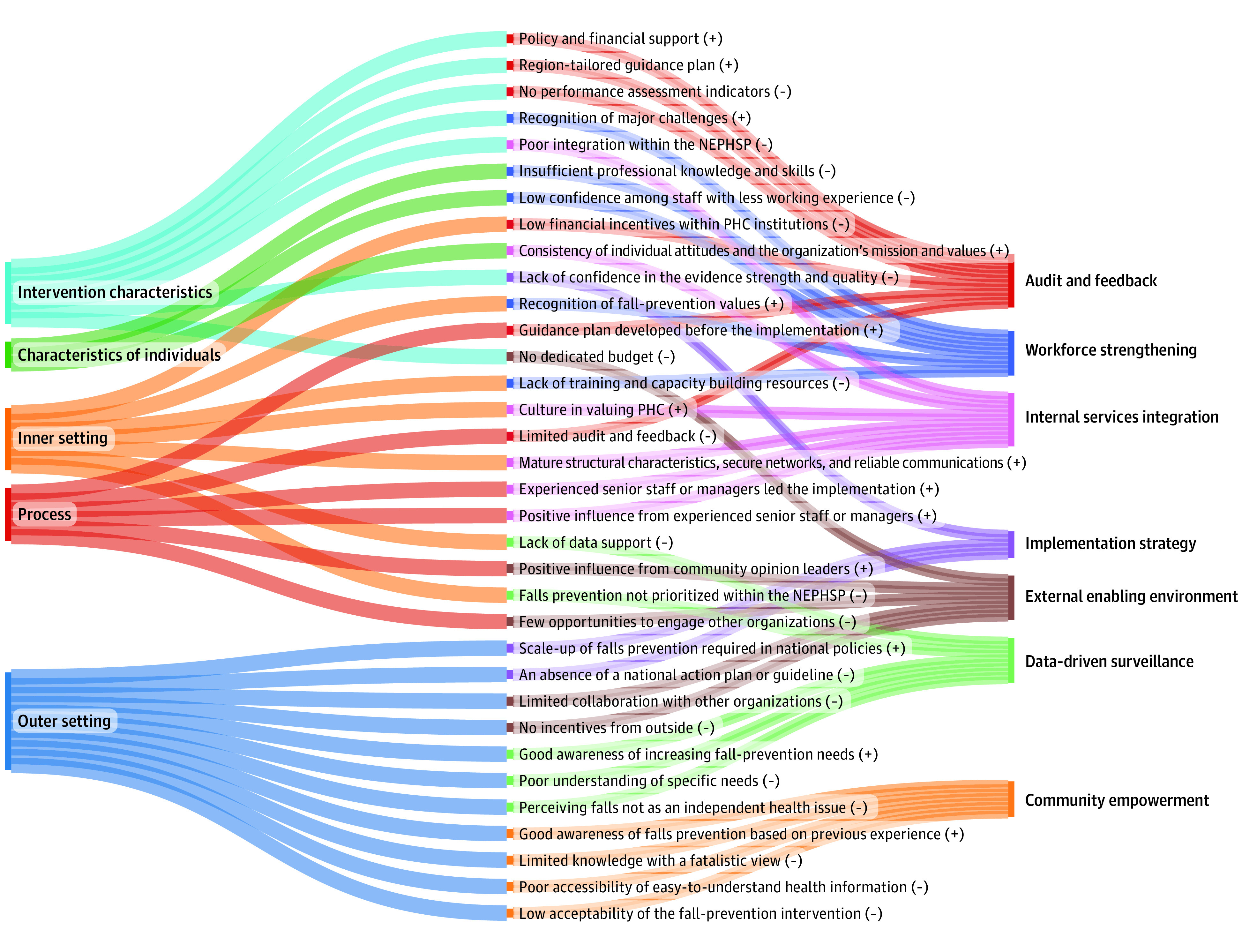
Identified Barriers and Facilitators in 7 Components of the Proposed Implementation Strategy NEPHSP indicates National Essential Public Health Service Package; PHC, primary health care; +, facilitators; −, barriers.

**Figure 3.**
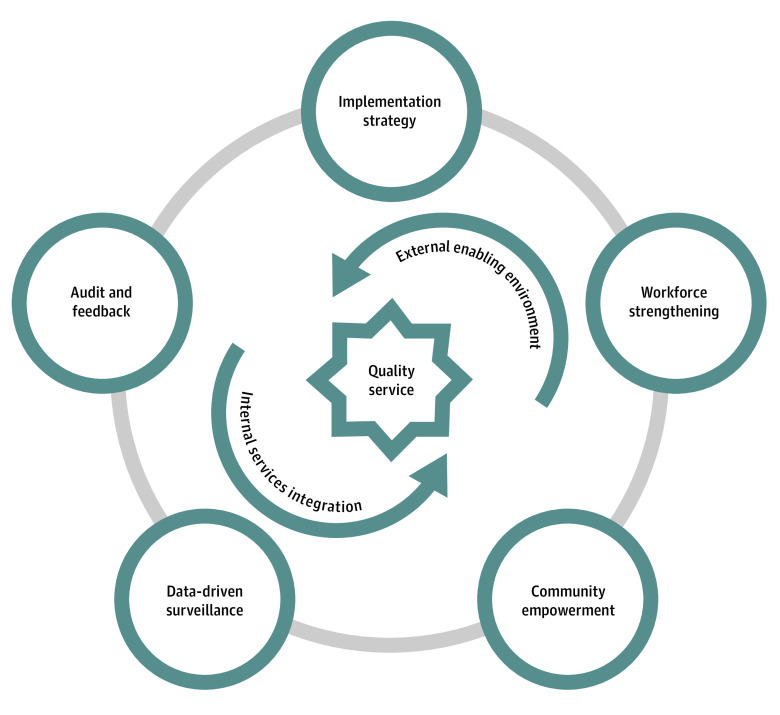
Visualization of the Framework Developed From This Study

A framework consisting of 7 interrelated strategies was developed to systematically respond to identified barriers (Figure 2 and Figure 3). Specifically, the data-driven surveillance echoes the poor understanding of the magnitude of falls and fall-prevention needs among older people, while audit and feedback respond to challenges resulting from the lack of performance assessment. The implementation strategy would potentially ease the concerns about the evidence strength and quality of current interventions. Likewise, workforce strengthening is likely to alleviate the anxiety centered on the capabilities of local stakeholders involved in the implementation process. We urge empowerment of the community to address the barriers to the needs and motivation of falls prevention among older people. We also recommend an internal services integration optimizing the resources inside the NEPHSP for falls prevention. Lastly, the external enabling environment underscores the potential mechanism of mobilizing external human resources and financial support.

## Discussion

This qualitative study was carried out by a multidisciplinary team with diverse research experience, to constantly reflect upon our attitudes, assumptions and positions in the interview process and analysis, to ensure that our understanding truly reflects the participants’ perspectives, roles, and concepts of the NEPHSP. This study identified the influencing factors to implementation of falls prevention programs for older people in the Chinese PHC settings. Clear recognition of the challenges and benefits of falls prevention, an adaptive regionally tailored guidance plan, and continuous governmental policy and financial support were the major facilitators, whereas the major barriers consisted of insufficient confidence in delivering interventions and poor understanding of the magnitude of falls, low recognition of the importance of falls prevention, limited multisectoral collaboration, and weak financial incentives. This study also developed 7 strategies to address these challenges.

The data-driven surveillance strategy could provide fundamental support to prioritize falls prevention under local jurisdiction. In China, the fragmentation of information systems in the design, interoperability, and data quality compromises the understanding of the epidemiology of falls. For example, the nonfatal outcomes of falls are separately recorded in the Chinese Chronic Disease and Risk Factor Surveillance program^20^ and Chinese National Injury Surveillance System,^21^ while fatal outcomes of falls are captured by the Chinese National Mortality Surveillance System.^22^ An integrated surveillance platform could routinely provide consistent analysis and interpretation of multiple data sources about the burden, cost, and risk factors of health issues over time. For example, the national data linkage infrastructure in Australia provides policy makers and researchers with comprehensive information of the health and well-being of the community.^23^ In Chinese PHC settings, armed with integrated data, service professionals could better prioritize and mobilize more resources for fall-prevention implementation.

A well-established audit and feedback strategy could improve professional practice and health care outcomes through an ongoing review of agreed performance indicators.^24^ However, the absence of performance indicators in the current audit and feedback loop could affect the quality of the implementation and health gains. Some key performance indicators, including the number of consultations per year, the number of older people in each education lecture, and the proportion of older people receiving the fall-risk screening, are therefore suggested additions. These process-oriented indicators could gauge the individual effort of PHC clinicians. To avoid the concern about the excessive pursuit of the number of service users over the service quality,^25^ some quality-oriented indicators could be included to encourage quality assurance fall-prevention practices (eg, the degree of satisfaction of older people toward the current intervention). It could also deepen service professionals’ understanding of older people’s views and perceptions, and the extent of their involvement in improving the quality of falls prevention programs.

A recent review reflected the low quality of evidence for the effectiveness of fall-prevention interventions in China,^4^ which were again identified as a barrier in this study. The implementation strategy aims to conduct robustly designed studies to evaluate the effectiveness of a regionally tailored fall-prevention program. In addition, a national task force of experts should be established to reach a consensus on the current and emerging evidence of implementation strategies in fall-prevention research. Based on this support, an evidence-informed national action plan or guideline can therefore be developed to articulate specific interventions with recommendation levels and minimum resources required. A similar technical package has been developed by the World Health Organization to support all concerned individuals and institutions to prevent and manage fall-related injuries in their work.^1^ This package provides implementation guidance on interventions for which implementation caveats feature strongly in the global evidence base.^1^

Health service coverage and outcomes are highly dependent on the quality of the health workforce.^26^ The lack of professional knowledge and skills of service professionals identified in this study could compromise effective fall-prevention implementation. To address this constraint, the workforce strengthening strategy recommends that various on-site and online training opportunities, for example, in-service training, seminars, and workshops, could be offered to junior and less highly trained PHC clinicians. These training resources should encompass essential parts of falls prevention (eg, communication skills, needs analysis, risk screening and management, and impact evaluation). The ultimate goal of training is to ensure that PHC clinicians have a clear understanding of key issues in falls prevention for older people and competency to perform their roles in implementation.

The current fall-prevention interventions often told older residents how to prevent falls, rather than listening and responding to their needs, which could be characterized as “a program was done for older people” not “a program was done with older people.” Consequently, it was not surprising that these interventions were viewed with apprehension and underused by many older people. The community empowerment strategy attempts to enable the transformation of older people being only end users into being engaged in self-determination of their communities. This change could boost their confidence and motivation to actively acquire more knowledge, skills, and resilience against fall-related injuries. Positive outcomes through promoting community empowerment, such as high retention in following services and a better sense of health gains, have been reported in previous studies.^27,28^ Of note, it would take a long time to show the benefits of community empowerment,^27^ but the time cost could be affordable in NEPHSP as long-term nationwide work.^5^

Presently, there are 5 other service items closely related to older people (eg, the health management service for patients with hypertension and type 2 diabetes).^29^ The information generated from these services could be shared to identify important fall-related risk factors for older people, such as fall history, poor vision, and the intake of multiple medications,^1,30^ and can be applied to provide personalized services in fall risk screening, prediction, and management. In addition, 2 service items related to health education^29^ (ie, health education service and health literacy promotion project), could facilitate the development of easy-to-understand fall-prevention information and the delivery of these materials to older people. Based on the experience shared in previous studies, the internal services integration strategy could have the potential to improve the cost-effectiveness, access, and uptake of fall-prevention intervention in older people.^31^

The external enabling environment strategy encourages identifying potential collaborators and forging partnerships with other like-minded agencies. As a cross-cutting health issue, the prevention of falls usually requires extensive resources beyond the capabilities of health sectors.^1,30^ For example, a safe outdoor environment with accessible transport infrastructure could encourage older people to maintain healthy levels of physical activity as they age, which would in turn reduce the risk of falls.^1,32^ The design, construction, and maintenance of these physical environments need intensive human resources and financial support from the department of transportation and urban construction rather than the health sectors. In addition, the strength of any group with an interest or responsibility for falls prevention could be leveraged to support the implementation. For example, civil society groups could be actively engaged in policy dialogue to address potential conflicts across different government departments in the implementation of falls prevention.^33^ Public-private partnerships could be introduced through the involvement of business and the private sector to raise additional funds to support resource-consuming interventions.^34^ All these collaborations could benefit the achievement of desired outcomes of the complex fall-prevention interventions.^35^

### Limitations

This study has some limitations. First, the findings should be transferred to other regions with caution due to the purposive sample method. However, readers could judge the transferability based on the details of the method. Second, all factors were qualitatively identified without causal inference. Further quantitative surveys should be conducted to triangulate these findings.

## Conclusions

This study identifies the facilitators and barriers to the implementation of falls prevention for older people in Chinese PHC settings, at 3 study sites, through a synthesis of qualitative interviews and discussions. Seven strategies were developed as a systematic response to foster the fall-prevention implementation for older people in Chinese PHC settings. These findings also have to the potential to provide valuable insights for other regions or countries experiencing similar challenges, within a rapidly aging population, to maximize the benefits of falls prevention for older people.

## Notes

Supplement. **eMethods.** Supplemental Methods **eTable 1.** The Facilitators and Barriers of Implementing Falls Prevention for Older People Across 5 Domains of the CFIR **eTable 2.** The Influencing Factors of Implementing Falls Prevention for Older People Across 5 Domains of the CFIR at the Construct-Level

## References

[1] World Health Organization. Step Safely-Strategies for Preventing and Managing Falls Across Life-course. World Health Organization; 2021.

[2] Leilei D, Pengpeng Y, Haagsma JA, . The burden of injury in China, 1990-2017: findings from the Global Burden of Disease Study 2017. Lancet Public Health. 2019;4(9):e449-e461. doi:10.1016/S2468-2667(19)30125-2 31493842 PMC6739690

[3] Ye P, Er Y, Wang H, . Burden of falls among people aged 60 years and older in mainland China, 1990-2019: findings from the Global Burden of Disease Study 2019. Lancet Public Health. 2021;6(12):e907-e918. doi:10.1016/S2468-2667(21)00231-0 34838197 PMC8646839

[4] Ye P, Liu Y, Zhang J, . Falls prevention interventions for community-dwelling older people living in mainland China: a narrative systematic review. BMC Health Serv Res. 2020;20(1):808. doi:10.1186/s12913-020-05645-0 32859186 PMC7456050

[5] Meng Q, Mills A, Wang L, Han Q. What can we learn from China’s health system reform? BMJ. 2019;365:l2349. doi:10.1136/bmj.l2349 31217222 PMC6598719

[6] Ministry of Health of China. National Essential Public Health Service Package Specification (2009 Edition). Accessed October 2, 2021. http://www.nhc.gov.cn/wjw/gfxwj/201304/b175eb09dfd240f6bae36d2fb67c8619.shtml

[7] The Leading Group Office of the Seventh National Census in Longhua District Shenzhen City. Bulletin of the Seventh National Census of Longhua District Shenzhen City. Accessed October 2, 2021. http://www.szlhq.gov.cn/attachment/0/794/794081/8858162.pdf

[8] The Government of Longhua District Shenzhen City. Statistical bulletin of national economic and social development of Longhua District, Shenzhen City in 2020 GDP. Accessed October 2, 2021. http://www.szlhq.gov.cn/attachment/0/794/794836/8860435.pdf

[9] The Leading Group Office of the Seventh National Census in Shijia Zhuang City. Bulletin of the Seventh National Census of Shijia Zhuang City. Accessed October 2, 2021. http://www.sjz.gov.cn/col/1596018184396/2021/05/31/1622426985480.html

[10] The Government of Chang’an District Shijia Zhuang City. Government Work Report of Chang'an District Shijia Zhuang City in 2021. Accessed October 2, 2021. http://www.sjzca.gov.cn/col/1586314439642/2021/04/16/1618542816323.html

[11] The Government of Beilun District Ningbo City. Bulletin of the Seventh National Census of Beilun District Ningbo City. Accessed October 2, 2021. http://www.bl.gov.cn/art/2021/5/17/art_1229055347_3730250.html

[12] Bureau of Statistics of Beilun District Ningbo City. Statistical Bulletin of National Economic and Social Development of Beilun District in 2020. Accessed October 2, 2021. http://www.bl.gov.cn/art/2021/3/1/art_1229054641_3708030.html

[13] Tong A, Sainsbury P, Craig J. Consolidated criteria for reporting qualitative research (COREQ): a 32-item checklist for interviews and focus groups. Int J Qual Health Care. 2007;19(6):349-357. doi:10.1093/intqhc/mzm042 17872937

[14] Damschroder LJ, Aron DC, Keith RE, Kirsh SR, Alexander JA, Lowery JC. Fostering implementation of health services research findings into practice: a consolidated framework for advancing implementation science. Implement Sci. 2009;4:50. doi:10.1186/1748-5908-4-50 19664226 PMC2736161

[15] Chinese Version of Consolidated Framework for Implementation Research. Accessed February 2, 2021. https://cfirguide.org/constructs/chinese/

[16] Saunders B, Sim J, Kingstone T, . Saturation in qualitative research: exploring its conceptualization and operationalization. Qual Quant. 2018;52(4):1893-1907. doi:10.1007/s11135-017-0574-8 29937585 PMC5993836

[17] Emliy CH, ed. Successful qualitative health research: a practical introduction. Routledge; 2020.

[18] Mansell I, Bennett G, Northway R, Mead D, Moseley L. The learning curve: the advantages and disadvantages in the use of focus groups as a method of data collection. Nurse Res. 2004;11(4):79-88. doi:10.7748/nr2004.07.11.4.79.c6217 15227901

[19] Waltz TJ, Powell BJ, Fernández ME, Abadie B, Damschroder LJ. Choosing implementation strategies to address contextual barriers: diversity in recommendations and future directions. Implement Sci. 2019;14(1):42. doi:10.1186/s13012-019-0892-4 31036028 PMC6489173

[20] Wang L, Zhou B, Zhao Z, . Body-mass index and obesity in urban and rural China: findings from consecutive nationally representative surveys during 2004-18. Lancet. 2021;398(10294):53-63. doi:10.1016/S0140-6736(21)00798-4 34217401 PMC7617101

[21] Duan L, Deng X, Wang Y, . The National Injury Surveillance System in China: a six-year review. Injury. 2015;46(4):572-579. doi:10.1016/j.injury.2014.12.013 25576399

[22] Liu S, Wu X, Lopez AD, . An integrated national mortality surveillance system for death registration and mortality surveillance, China. Bull World Health Organ. 2016;94(1):46-57. doi:10.2471/BLT.15.153148 26769996 PMC4709796

[23] Young A, Flack F. Recent trends in the use of linked data in Australia. Aust Health Rev. 2018;42(5):584-590. doi:10.1071/AH18014 30145995

[24] Ivers N, Jamtvedt G, Flottorp S, . Audit and feedback: effects on professional practice and healthcare outcomes. Cochrane Database Syst Rev. 2012;(6):CD000259. doi:10.1002/14651858.CD000259.pub3 22696318 PMC11338587

[25] Yuan B, Balabanova D, Gao J, Tang S, Guo Y. Strengthening public health services to achieve universal health coverage in China. BMJ. 2019;365:l2358. doi:10.1136/bmj.l2358 31227480 PMC6598722

[26] World Health Organization. Health workforce 2030: towards a global strategy on human resources for health. World Health Organization; 2015.

[27] World Health Organization. Regional Office for the Eastern Mediterranean. (‎2003)‎. Community empowerment for health and development. World Health Organization. Regional Office for the Eastern Mediterranean. Accessed October 2, 2021. https://apps.who.int/iris/handle/10665/201123

[28] Haldane V, Chuah FLH, Srivastava A, . Community participation in health services development, implementation, and evaluation: a systematic review of empowerment, health, community, and process outcomes. PLoS One. 2019;14(5):e0216112. doi:10.1371/journal.pone.0216112 31075120 PMC6510456

[29] National Health Commission of China. National Essential Public Health Service Package Specification (2019 Edition). Accessed October 2, 2021. http://www.gov.cn/fuwu/2019-09/06/content_5427746.htm

[30] World Health Organization. WHO Global Report on Falls Prevention in Older Age. World Health Organization; 2007.

[31] Heyeres M, McCalman J, Tsey K, Kinchin I. The complexity of health service integration: a review of reviews. Front Public Health. 2016;4:223. doi:10.3389/fpubh.2016.00223 27800474 PMC5066319

[32] Ye P, Jin Y, Er Y, . A scoping review of national policies for healthy ageing in mainland China from 2016 to 2020. Lancet Reg Health West Pac. 2021;12:100168. doi:10.1016/j.lanwpc.2021.100168 34527965 PMC8356098

[33] Beinare D, McCarthy M. Civil society organisations, social innovation and health research in Europe. Eur J Public Health. 2012;22(6):889-893. doi:10.1093/eurpub/ckr152 22117053

[34] Tabrizi JS, Azami-Aghdash S, Gharaee H. Public-private partnership policy in primary health care: a scoping review. J Prim Care Community Health. 2020;11:2150132720943769. doi:10.1177/2150132720943769 32842863 PMC7453464

[35] Kuruvilla S, Hinton R, Boerma T, ; PMNCH Multisectoral Collaboration Study Group. Business not as usual: how multisectoral collaboration can promote transformative change for health and sustainable development. BMJ. 2018;363:k4771. doi:10.1136/bmj.k4771 30530519 PMC6282730

