# Supplementary material for: Perceptions of Facilitators and Barriers to Implementation of Falls Prevention Programs in Primary Health Care Settings in China
Source: JAMA Netw Open. 2022 Aug 26;5(8):e2228960. doi: 10.1001/jamanetworkopen.2022.28960 (PMC9419020; doi:10.1001/jamanetworkopen.2022.28960)
Supplement: Supplement. — eMethods. Supplemental Methods eTable 1. The Facilitators and Barriers of Implementing Falls Prevention for Older People Across 5 Domains of the CFIR eTable 2. The Influencing Factors of Implementing Falls Prevention for Older People Across 5 Domains of the CFIR at the Construct-Level [file jamanetwopen-e2228960-s001.pdf]

Supplemental Online Content

Ye P, Jin Y, Er Y, et al. Perceptions of facilitators and barriers to implementation of falls prevention programs in primary health care settings in China. *JAMA Netw Open.* 2022;5(8):e2228960. doi:10.1001/jamanetworkopen.2022.28960

eMethods. Supplemental Methods

eTable 1. The Facilitators and Barriers of Implementing Falls Prevention for Older People Across 5 Domains of the CFIR

eTable 2. The Influencing Factors of Implementing Falls Prevention for Older People Across 5 Domains of the CFIR at the Construct-Level

This supplemental material has been provided by the authors to give readers additional information about their work.

© 2022 Ye P et al. *JAMA Network Open.*

eMethods.

1. The background of three study sites

The three study sites were purposively selected based on their good performance in providing health services for older residents over the past five years and their willingness to participate.

In China, most provincial health care policies were formulated from the national policies with minimum modifications to the local context. This study primarily sought to identify the factors associated with falls prevention, as part of the NEPHSP, at the primary health care settings. Provincial health policies related to the NEPHSP are relatively homogenous regardless of the locations. Health care providers are tasked to follow the same policies to implement the falls prevention program at the primary health care settings.

2. The sampling method

A combination of purposive sampling and snowball sampling was adopted to recruit participants. For service providers, to maintain arms-length recruitment in the study, a list of potential participants was developed for each study site, based on the researchers' existing contact networks and the publicly available information from the organisational websites, prior to the recruitment. Emails were sent to all potential participants to confirm their willingness to participate. Snowball sampling was used during the interview to identify additional participants. Older residents were recruited by their local PHC providers.

3. The brief description of CFIR

CFIR is a meta-theoretical framework, which has been widely used to guide the identification of barriers and facilitators and build the implementation knowledge base across multiple studies and settings. It consists of 36 constructs within five domains: (1) intervention characteristics (i.e., the source, evidence and cost of falls prevention); (2) outer setting (i.e., the needs of community-dwelling older people for falls prevention, external policy and incentives for falls prevention); (3) inner setting (i.e., diverse characteristics of implementation organization); (4) characteristics of individuals (i.e., knowledge, personal attributes of stakeholders involved in the implementation); (5) process (i.e., planning and engaging in the implementation process). For each stakeholder group, the interview guide was tailored to better reflect their role and nature in the implementation of falls prevention for older people in the primary health care settings [1].

4. The brief introduction of ERIC

The Expert Recommendations for Implementing Change (ERIC) was developed to address two major limitations of the published literature: lack of conceptual clarity with regard to implementation strategies and insufficient guidance about how to select appropriate strategies for implementing a particular evidence-based programs and practices in a particular context. The ERIC process involved a four-stage sequential mixed methods design. Stages 1 and 2 were used to establish expert consensus on a common nomenclature for implementation science. Stages 3 and 4 build upon the earlier stages and were used to develop expert recommendations regarding how to best match discrete implementation strategies to high priority implementation scenarios. The ERIC tool is a novel method of selecting implementation strategies for use within specific contexts and can be used to identify priority implementation strategies [2]. In our research study, the barriers were mapped to the ERIC tool to inform recommendation for successful implementation."

5. The external experts panel

In the strategy development, seven external experts, who were members of the national injury prevention and control committee, were consulted. All experts were senior staff members in their organisations with professor or equivalent titles, including one gerontologist, two general practitioners, one injury epidemiologist, two professionals in health policy and systems, and one expert in health communication.

Reference

1. Damschroder LJ, Aron DC, Keith RE, Kirsh SR, Alexander JA, Lowery JC. Fostering implementation of health services research findings into practice: a consolidated framework for advancing implementation science. *Implement Sci*. 2009;4:50. doi: 10.1186/1748-5908-4-50.

© 2022 Ye P et al. *JAMA Network Open.*

2. Waltz TJ, Powell BJ, Chinman MJ, Smith JL, Matthieu MM, Proctor EK, Damschroder LJ, Kirchner JE. Expert Recommendations for Implementing Change (ERIC): protocol for a mixed methods study. *Implement Sci*. 2014;9:39. doi: 10.1186/1748-5908-9-39.

6. Interview guide

Interview guide
Health administrators

Beginning: Go through information sheet and obtain written informed consent

1. Introduction of the interviewer
2. Introduce research purpose
3. Introduce the scope and the time of the interview
4. Introduce participants' rights regarding the withdrawal from the interview
5. Confidentiality and use of data
6. Obtain written informed consent

Indicative interview questions

1. Could you tell us something below about your organization?
   a. The norm and values
   b. The development and current situation
   c. The social networks and formal/informal communication
   d. The conflict coordination of team members
   e. The self-worth cognition and evaluation of team members
   f. The innovative ability and willingness of team members

2. How do you perceive the needs of falls prevention in your area?
   a. Is falls prevention an urgent need for older people?
   b. Is falls prevention a key priority for primary health care providers?

3. What provincial/city-level/district or county-level policies have been formulated to focus on the falls prevention for older people in your area?
   a. If any, how successful have these policies been in addressing the falls burden of older people?
   b. If any, have these policies been integrated into the delivery of health management service for older people?

4. What interventions have been taken to prevent falls for older people in your area?

5. Could you tell us something below about the formulation of interventions that have been taken to prevent falls for older people in your area?
   a. Are these interventions uniformly formulated by higher authorities, or designed within your organization?
   b. Do your staff have different views on these interventions?
   c. Is there any extrinsic incentive for these interventions? For example, goal-sharing awards, performance reviews, promotions, and raises in salary, increased stature or respect.
   d. Is there any access to digestible information and knowledge about the intervention and how to incorporate it into work tasks?
   e. Is there any tangible and immediate indicators of organizational commitment to its decision to implement these interventions?

6. Could you tell us something below about the implementation of interventions that have been taken to prevent falls for older people in your area?
   a. Is there any plan for the implementation of these interventions developed in advance?
   b. Are these interventions carried out or accomplished according to the plan?
   c. Is there any quantitative and qualitative feedback about the progress and quality of implementation accompanied with regular personal and team debriefing about progress and experience?

© 2022 Ye P et al. *JAMA Network Open.*

   d. What are the commitment, involvement, and accountability of leaders and managers in your organization with the implementation?
   e. What many resources are dedicated for implementation and on-going operations including money, training, education, physical space, and time?

7. Could you tell us something below about the evaluation of interventions that have been taken to prevent falls for older people in your area?
   a. Is there sufficient time and space for reflective thinking and evaluation?
   b. Are goals clearly communicated, acted upon, and fed back to your staff and alignment of that feedback with goals?
   c. How these interventions align with your staff's own norms, values, and perceived risks and needs?
   d. How these interventions fit with existing workflows and system within your organization?

8. Could you tell us something below about key individuals involved in these implementations?
   a. Is there any combined strategy to attract and involve appropriate individuals in the implementation of the intervention?
   b. Is there any individual in your organization who has formal or informal influence on the attitudes and beliefs of their colleagues with respect to implementing the intervention?
   c. Is there any individual from your organization who has been formally appointed with responsibility for implementing an intervention as coordinator, project manager, team leader, or other similar role?
   d. Is there any individual who dedicates himself/herself to supporting, marketing, and 'driving through' an implementation overcoming indifference or resistance that the intervention may provoke in your organization?
   e. Is there any individual who is affiliated with an outside entity who formally influences or facilitates intervention decisions in a desirable direction?

9. Could you tell us something below about any external organization that might be engaged in the same or similar interventions in your area?
   a. Could you describe what does this/these organization(s) work in the field of falls prevention for older people?
   b. Could you describe how this/ these organisation(s) work?
   c. Are your organization networked with those external organization to implement falls prevention interventions?
   d. Would there be any competitive pressure from external organizations?

10. How successful is the implementation of falls prevention interventions for older people in your area?
   a. What works well?
   b. What are the barriers to it functioning better?
   c. What changes do you think are needed to improve it?
   d. Will you consider to spread these interventions and how?

Discussion guide

Primary health care providers and CDC staff

Beginning: Go through information sheet and obtain written informed consent

Introduction of the interviewer

1. Introduce research purpose
2. Introduce the scope and the time of the interview
3. Introduce participants' rights regarding the withdrawal from the interview
4. Confidentiality and use of data
5. Obtain written informed consent

Indicative interview questions

1. Is the falls prevention one of key priorities in health management service for older people in your area?
    a. If not, why?
    b. If yes, why?

2. Have you been trained in the field of falls prevention for older people?
    a. If yes, who provides the training and how often?
    b. If yes, who in the team accesses this training?
    c. If yes, what supervisory practices are in place?
    d. If yes, what continuing education opportunities are there?
    e. If yes, how could training be improved?
    f. If no, have you learned about the falls prevention for older people in other ways? e.g., from relevant articles, reports, books or videos programs?
    g. If no, do you want to participate in similar trainings in the future?
    h. If no, what factors could keep you from participating in similar trainings?
    i. If no, what kind of information do you want to get in the training?

3. Can you talk something about following items related to the falls prevention for older people in your area?
    a. Epidemiological status: do you know any falls burden in your area, e.g., incidence, prevalence, mortality, disability and falls-related injury
    b. Risk factors: do you know any risk factor of falls in older population?
    c. Risk assessment: do you know any method or tool used to assess the risk of falls for older people?
    d. Prevention measures: do you know any measure that could be adopted by older people to prevent falls?
    e. Guidelines: do you know any domestic or international guideline related to falls prevention?
    f. Treatment: do you know any treatment for older people after falls?
    g. Rehabilitation: do you know any rehabilitation for older people after falls?

4. Do you understand the needs of older residents in the community for falls prevention?
    a. Are there any barriers to meet these needs? If yes, how to overcome these barriers?
    b. Are there any facilitators to meet these needs? If yes, how to leverage these facilitators?

5. What interventions have been taken to prevent fall prevention for older residents in your area?
    a. Are these interventions uniformly formulated by higher authorities, or are they designed within your organization?

6. What is the desired goal of these interventions?

7. Do you think these interventions can achieve the desired goal?

8. Is the implementation of these interventions based on existing research evidence?
    a. If yes, what research evidence is it based on, such as articles, guidelines, reports from authoritative organizations, professional staff?
    b. If not, is it only based on the staffs' personal experience and knowledge?

9. If there are different interventions, is there any difference in their effects of falls prevention?

10. Have these interventions been adapted, tailored, refined, or reinvented to meet local needs?

© 2022 Ye P et al. *JAMA Network Open.*

11. Have these interventions been tested on a small scale in the organization, and to be able to reverse course (undo implementation) if warranted.

12. Is there any difficulty of implementation these interventions?
    a. If yes, what are these difficulties?

13. How the intervention is bundled, presented, and assembled?

14. What is the cost of the intervention and cost associated with implementing that intervention including investment, supply, and opportunity costs?

15. What is your attitudes toward and value placed on these interventions?

16. Are you familiar with the fact and principle related to these interventions?

17. Do you believe that you are capable to execute courses of action to achieve the goal of these interventions?

18. How long have you worked in this organization?

19. How long have you been implementing these interventions?

20. How do you perceive the organization and their relationship?

21. Could you tell us something about your personal traits such as tolerance of ambiguity, intellectual ability, motivation, values, competence, capacity, and learning style?

© 2022 Ye P et al. *JAMA Network Open.*

Discussion guide
Older people

Beginning: Go through information sheet and obtain written informed consent

1. Introduction of the interviewer
2. Introduce research purpose
3. Introduce the scope and the time of the interview
4. Introduce participants' rights regarding the withdrawal from the interview
5. Confidentiality and use of data
6. Obtain written informed consent

Interview questions guides

1. Today we are talking about the implementation of falls prevention in health management service for older people. Can you tell me what you know about the health management service?
    a. Is the health management service a concern in your community?
    b. What should be included?
    c. What has been your personal experience when you accepting this service?
    d. Is the health management service necessary for a healthier life?

2. Can you tell me what you know about the falls prevention?
    a. What is the fall?
    b. Have you experienced any fall before? In the last 12 months?
    c. Are you worried about falling?
    d. Are you afraid of falling?
    e. Is the falls prevention a concern in your daily life?
    f. Do you know any risk factor of falls?
    g. Do you know any measure to prevent falls?
    h. Do you need the falls prevention guidance?

3. Have you received any falls prevention outside primary health care institutions in your area, including any written material, TV program, lecture, poster, and brochure?
    a. If yes, who provided the falls prevention guidance?
    b. If yes, what kind of falls prevention guidance did you learn about?
    c. If no, do you want to receive any falls prevention guidance?
    d. If no, what do you want to know in this guidance?

4. Have you received any falls prevention service when you receiving the health management service in primary health care institutions?
    a. If yes, who provided you with the falls prevention guidance?
    b. If yes, did they inform you actively or you ask them to tell you something about the falls prevention?
    c. If yes, did you know the reason why they informed you about the falls prevention guidance or you want them to tell you?
    d. If yes, what was the delivery model of falls prevention guidance you received, e.g., verbal, paper-based, video, regular lecture, and other forms?
    e. If yes, what kind of falls prevention guidance did you learn about?
    f. If yes, do you feel you get enough time to discuss the things you need to when you see your care provider?
    g. If yes, do you experience any challenges understanding the information you receive?
    h. If yes, was this guidance useful in your daily life in your perspective?
    i. If no, did you ask primary health care providers any question about the falls prevention?
    j. If no, do you want to know the falls prevention guidance from primary health care providers when you receiving health management service?
    k. If no, what the delivery model of falls prevention guidance do you prefer, e.g., verbal, paper-based, video, regular lecture, and other forms?
    l. If no, what kind of falls prevention guidance do you want to learn about?

5. What things are needed to make it easier for you to get good falls prevention service?
    a. What do you think are the challenges for primary health care providers to deliver their work effectively?

© 2022 Ye P et al. *JAMA Network Open.*

    b. If you were in charge of your health service what would you do differently?

6. Do community members/leaders play a role in health management services in your community?
    a. Can you describe what activities are happening in your community to reduce harm from falls?

© 2022 Ye P et al. *JAMA Network Open.*

7. Results of matching tool

Implementation strategies as recommended by the CFIR-ERIC matching tool

ERIC Strategies	Cumulative Percent	Evidence Strength & Quality	Design Quality & Packaging	Cost	Patient Needs & Resources	Cosmopolitanism	External Policy & Incentives	Tension for Change	Compatibility	Relative Priority	Organizational Incentives & Rewards	Goals and Feedback	Leadership Engagement	Available Resources	Access to knowledge & information	Knowledge & Beliefs about the Intervention	Self-efficacy	External Change Agents	Reflecting & Evaluating
Identify and prepare champions	**403%**	41%	15%	12%	5%	15%	22%	48%	21%	18%	25%	12%	41%	4%	24%	40%	30%	22%	8%
Conduct local consensus discussions	**371%**	41%	26%	4%	29%	15%	22%	43%	41%	46%	8%	18%	27%	0%	10%	12%	0%	19%	8%
Conduct educational meetings	**347%**	47%	22%	12%	10%	12%	15%	17%	10%	7%	0%	21%	9%	0%	79%	56%	15%	11%	4%
Alter incentive/allowance structures	**324%**	3%	0%	44%	10%	0%	41%	22%	10%	39%	71%	15%	32%	17%	0%	16%	4%	0%	0%
Build a coalition	**306%**	6%	0%	4%	14%	62%	33%	9%	21%	18%	17%	15%	18%	17%	3%	16%	0%	44%	8%
Capture and share local knowledge	**303%**	25%	15%	4%	10%	23%	26%	13%	14%	14%	8%	12%	9%	22%	31%	24%	19%	11%	24%
Assess for readiness and identify barriers and facilitators	**300%**	13%	7%	16%	33%	15%	4%	35%	34%	36%	13%	6%	14%	13%	7%	20%	11%	11%	12%
Inform local opinion leaders	**284%**	38%	19%	12%	0%	15%	22%	39%	3%	14%	17%	18%	18%	0%	7%	28%	4%	22%	8%
Conduct local needs assessment	**265%**	3%	15%	4%	57%	12%	7%	43%	21%	32%	8%	6%	14%	0%	3%	24%	0%	11%	4%
Create a learning collaborative	**261%**	16%	7%	8%	0%	31%	15%	9%	14%	4%	13%	12%	5%	9%	45%	16%	30%	22%	8%
Audit and provide feedback	**247%**	13%	4%	8%	5%	0%	0%	17%	7%	14%	21%	61%	5%	0%	3%	4%	22%	7%	56%
Involve executive boards	**246%**	6%	4%	20%	5%	23%	41%	13%	3%	11%	13%	0%	45%	17%	0%	0%	0%	37%	8%
Access new funding	**240%**	3%	4%	72%	0%	4%	7%	0%	3%	11%	38%	3%	9%	78%	0%	8%	0%	0%	0%
Promote adaptability	**230%**	3%	48%	16%	14%	0%	0%	17%	45%	18%	4%	9%	9%	4%	7%	16%	11%	7%	0%
Develop educational materials	**219%**	28%	33%	0%	10%	4%	4%	0%	3%	7%	0%	9%	0%	4%	59%	36%	19%	4%	0%
Conduct educational outreach visits	**218%**	34%	15%	4%	5%	23%	0%	4%	0%	14%	4%	12%	9%	0%	28%	28%	22%	11%	4%
Use advisory boards and workgroups	**217%**	9%	19%	0%	29%	35%	15%	13%	3%	7%	4%	12%	18%	4%	0%	8%	7%	33%	0%
Obtain and use patients/consumers and family feedback	**217%**	6%	30%	4%	76%	0%	0%	9%	10%	7%	13%	9%	14%	0%	0%	4%	4%	4%	28%
Facilitation	**209%**	0%	7%	8%	0%	12%	4%	0%	24%	14%	4%	18%	18%	4%	10%	20%	22%	22%	20%
Develop and implement tools for quality monitoring	**205%**	6%	30%	0%	14%	0%	11%	9%	3%	7%	21%	27%	9%	0%	0%	0%	4%	4%	60%
Develop a formal implementation blueprint	**203%**	0%	15%	8%	5%	4%	7%	13%	3%	14%	8%	42%	23%	4%	14%	4%	11%	11%	16%
Involve patients/consumers and family members	**201%**	13%	19%	0%	71%	4%	11%	22%	10%	18%	4%	9%	9%	0%	3%	0%	4%	0%	4%
Tailor strategies	**192%**	6%	15%	12%	14%	0%	11%	13%	38%	14%	17%	0%	5%	9%	0%	12%	11%	4%	12%
Use an implementation adviser	**174%**	6%	15%	4%	5%	8%	4%	13%	10%	7%	0%	12%	18%	13%	14%	0%	7%	26%	12%
Develop academic partnerships	**174%**	25%	4%	4%	5%	50%	11%	4%	0%	0%	4%	3%	0%	4%	10%	12%	7%	26%	4%
Visit other sites	**170%**	13%	4%	16%	0%	38%	7%	13%	10%	4%	4%	0%	5%	9%	14%	12%	15%	7%	0%
Facilitate relay of clinical data to providers	**170%**	6%	7%	0%	10%	0%	4%	22%	3%	7%	4%	36%	5%	0%	10%	12%	7%	0%	36%
Conduct cyclical small tests of change	**167%**	3%	11%	8%	10%	0%	4%	4%	38%	4%	13%	3%	0%	13%	3%	12%	26%	4%	12%
Identify early adopters	**167%**	22%	11%	8%	0%	4%	7%	13%	10%	7%	13%	6%	9%	0%	10%	20%	19%	7%	0%
Fund and contract for clinical innovation	**166%**	0%	4%	28%	0%	0%	15%	9%	10%	11%	21%	0%	18%	39%	3%	4%	4%	0%	0%
Purposely reexamine the implementation	**160%**	6%	22%	0%	5%	4%	11%	4%	28%	4%	4%	12%	9%	4%	0%	4%	0%	15%	28%
Organize clinician implementation team meetings	**158%**	3%	4%	0%	0%	0%	0%	9%	14%	4%	8%	36%	0%	9%	14%	4%	11%	15%	28%
Provide ongoing consultation	**158%**	9%	7%	0%	5%	0%	0%	4%	3%	14%	4%	15%	14%	0%	17%	4%	41%	11%	8%
Distribute educational materials	**149%**	31%	19%	0%	5%	0%	0%	13%	0%	4%	0%	3%	0%	0%	55%	16%	4%	0%	0%
Promote network weaving	**146%**	0%	0%	0%	0%	50%	11%	4%	0%	4%	8%	6%	9%	9%	10%	12%	4%	19%	0%
Conduct ongoing training	**145%**	6%	4%	0%	0%	0%	4%	0%	0%	4%	0%	9%	0%	9%	38%	12%	41%	4%	16%
Obtain formal commitments	**139%**	0%	0%	0%	0%	19%	15%	0%	0%	14%	13%	12%	27%	13%	0%	0%	0%	26%	0%
Provide local technical assistance	**138%**	3%	4%	4%	5%	4%	7%	0%	14%	0%	0%	12%	5%	0%	24%	0%	22%	19%	16%
Increase demand	**134%**	0%	4%	12%	10%	0%	0%	13%	0%	29%	8%	0%	27%	4%	0%	20%	0%	7%	0%
Model and simulate change	**129%**	3%	11%	20%	0%	8%	4%	13%	3%	0%	0%	9%	14%	0%	7%	4%	33%	0%	0%
Recruit, designate and train for leadership	**127%**	3%	0%	4%	0%	15%	0%	4%	0%	11%	21%	18%	23%	4%	3%	4%	4%	4%	8%
Develop and organize quality monitoring systems	**118%**	3%	0%	4%	0%	0%	15%	9%	3%	0%	4%	24%	5%	0%	0%	0%	7%	4%	40%
Develop resource sharing agreements	**115%**	0%	0%	32%	0%	31%	0%	0%	3%	0%	4%	0%	0%	26%	3%	0%	4%	11%	0%
Place innovation on fee for service lists/formularies	**107%**	6%	0%	24%	0%	0%	19%	4%	3%	7%	8%	0%	14%	17%	0%	0%	0%	0%	4%
Mandate change	**105%**	0%	0%	8%	0%	0%	15%	13%	3%	32%	13%	0%	14%	0%	0%	4%	4%	0%	0%
Make training dynamic	**103%**	6%	11%	0%	0%	0%	0%	0%	3%	7%	8%	6%	9%	0%	10%	0%	41%	0%	0%
Stage implementation scale up	**100%**	3%	4%	8%	0%	0%	4%	4%	10%	7%	4%	0%	0%	13%	3%	20%	15%	0%	4%
Use data experts	**96%**	6%	4%	12%	0%	4%	4%	17%	0%	7%	0%	3%	0%	0%	0%	4%	4%	4%	28%
Use other payment schemes	**93%**	0%	0%	20%	0%	0%	15%	0%	0%	7%	25%	0%	5%	22%	0%	0%	0%	0%	0%
Prepare patients/consumers to be active participants	**83%**	0%	0%	0%	48%	0%	0%	9%	3%	0%	4%	3%	5%	0%	0%	0%	0%	4%	8%
Work with educational institutions	**81%**	16%	11%	4%	0%	19%	4%	0%	3%	0%	4%	0%	0%	4%	7%	4%	0%	0%	4%
Shadow other experts	**80%**	3%	4%	0%	0%	4%	0%	4%	3%	0%	0%	0%	0%	0%	21%	4%	33%	4%	0%
Use train the trainer strategies	**79%**	3%	0%	0%	0%	8%	4%	0%	0%	0%	4%	3%	0%	9%	10%	4%	15%	7%	12%
Make billing easier	**78%**	0%	0%	32%	0%	0%	7%	0%	0%	4%	8%	0%	5%	22%	0%	0%	0%	0%	0%
Develop disincentives	**77%**	0%	0%	16%	0%	0%	7%	9%	0%	7%	8%	3%	23%	0%	0%	0%	0%	0%	4%
Provide clinical supervision	**74%**	3%	0%	0%	5%	0%	0%	0%	10%	0%	8%	15%	0%	0%	17%	0%	11%	0%	4%
Intervene with patients/consumers to enhance uptake & adherence	**71%**	3%	7%	4%	24%	0%	4%	0%	3%	4%	4%	0%	14%	0%	0%	0%	0%	0%	4%
Create or change credentialing and/or licensure standards	**69%**	3%	0%	4%	0%	4%	19%	4%	0%	14%	17%	0%	0%	4%	0%	0%	0%	0%	0%
Change physical structure and equipment	**63%**	0%	0%	4%	0%	0%	0%	0%	7%	0%	4%	0%	0%	48%	0%	0%	0%	0%	0%
Revise professional roles	**60%**	0%	0%	0%	0%	0%	4%	0%	10%	0%	13%	9%	5%	9%	3%	0%	0%	4%	4%
Use mass media	**59%**	6%	0%	4%	0%	8%	15%	4%	3%	7%	0%	0%	0%	0%	3%	8%	0%	0%	0%
Change record system	**57%**	0%	0%	0%	5%	4%	4%	4%	0%	7%	0%	9%	0%	4%	0%	0%	4%	0%	16%
Alter patient/consumer fees	**53%**	0%	0%	20%	0%	0%	7%	0%	0%	0%	4%	0%	0%	22%	0%	0%	0%	0%	0%
Centralize technical assistance	**53%**	3%	4%	0%	0%	4%	0%	0%	10%	0%	0%	9%	5%	0%	3%	0%	11%	4%	0%
Develop an implementation glossary	43%	0%	4%	4%	0%	4%	4%	0%	0%	0%	0%	9%	5%	0%	7%	4%	0%	4%	0%
Change accreditation or membership reqs	43%	0%	0%	4%	0%	8%	15%	0%	0%	4%	8%	0%	5%	0%	0%	0%	0%	0%	0%
Change liability laws	42%	0%	0%	0%	0%	0%	19%	4%	7%	0%	8%	0%	0%	0%	0%	0%	0%	4%	0%
Use capitated payments	41%	0%	0%	16%	0%	0%	0%	0%	0%	4%	4%	0%	5%	9%	0%	0%	0%	0%	4%
Create new clinical teams	32%	0%	0%	0%	10%	0%	0%	0%	7%	0%	0%	0%	0%	4%	0%	0%	7%	4%	0%
Start a dissemination organization	31%	0%	4%	0%	5%	8%	0%	0%	0%	0%	0%	3%	0%	0%	0%	4%	0%	4%	4%
Use data warehousing techniques	24%	3%	0%	0%	5%	0%	0%	0%	0%	0%	0%	0%	5%	0%	0%	0%	0%	0%	12%
Change service sites	15%	3%	0%	0%	0%	0%	4%	0%	3%	0%	0%	0%	0%	4%	0%	0%	0%	0%	0%
Remind clinicians	15%	0%	0%	0%	0%	0%	0%	0%	0%	4%	0%	3%	5%	0%	3%	0%	0%	0%	0%

Note: CFIR = Consolidated Framework for Implementation Research; ERIC = Expert Recommendations for Implementing Change

© 2022 Ye P et al. *JAMA Network Open.*

eTable 1. The facilitators and barriers of implementing falls prevention for older people across 5 domains of the CFIR

CFIR domains	Facilitators	Barriers
**Intervention characteristics**	• Continuous policy and financial support from central and local governments.<br>• Region-tailored guidance plan was available and refined on an annual basis<br>• Service providers recognized the perceived major challenges of implementing the intervention.	• Service providers hesitated to provide current interventions due to the lack of confidence in the evidence strength and quality.<br>• No performance assessment indicators defined.<br>• Poor integration within the health management for older people and with other service items in the NEPHSP.<br>• No dedicated budget allocated to falls prevention for older people.
**Outer setting**	• Service providers realized that more local older residents would need fall-prevention service.<br>• Older people with prior experience of fall-related injuries had a good awareness of falls prevention.<br>• National health policies required the scale-up of falls prevention for older people.	• Service providers had poor understanding of specific needs for falls prevention among local older residents, while PHC providers perceived falls not as an independent health issue.<br>• Older people had limited knowledge of falls prevention and traditionally viewed falls as an inevitable accident in life.<br>• Illiterate older people had poor accessibility of easy-to-understand health educational information on falls prevention. In addition, older people had a weak willingness to accept fall-prevention intervention due to the unforeseen health gain from the current service.<br>• Limited collaboration with other government departments, civil societies, private sectors, and academic institutions.<br>• An absence of a national action plan or guideline for falls prevention for community-dwelling older people.<br>• No financial incentives from outside of the organizations provided to the service providers.
**Inner setting**	• The organizations of service providers shared central elements of culture in valuing PHC and had mature structural characteristics, secure networks and reliable communications.<br>• Service providers recognized the perceived values of falls prevention to improve older people's health.	• Service providers did not realize the growing burden of falls among local older people due to the lack of data support, and failed to prioritize falls prevention within the NEPHSP.<br>• Low financial incentives within PHC institutions.<br>• The lack of training and capacity building resources provided to the PHC providers.
**Characteristics of individuals**	• Most service providers' positive attitudes towards the implementation of fall-prevention intervention were consistent with their organization's mission and values.	• Service providers were not well equipped with professional knowledge and skills to implement fall-prevention interventions.<br>• PHC providers with less working experience had low confidence in implementing fall-prevention interventions based on their own capabilities

© 2022 Ye P et al. *JAMA Network Open.*

**Process**	• Region-tailored guidance plan was well-developed before the implementation.<br>• Experienced senior staff or managers led the implementation of the intervention and positively influenced the attitudes and beliefs of their colleagues.<br>• Community opinion leaders had a positive influence on the implementation of falls prevention for older people	• Few opportunities provided to civil societies, private sectors, and academic institutions in participating the planning and implementation of fall-prevention intervention.<br>• Limited audit and feedback.

© 2022 Ye P et al. *JAMA Network Open.*

eTable 2. The influencing factors of implementing falls prevention for older people across 5 domains of the CFIR at the construct-level

Domains and Constructs	Facilitators	Barriers	Neutral Factors
**I. Intervention characteristics**			
A Intervention Source	1. A mandatory requirement in the NEPHSP as a long-term nationwide work led by governments		
B Evidence Strength & Quality		1. Service providers lacked confidence in the evidence strength and quality for current interventions predominated by education measures	
C Relative advantage			1. No alternative plan or solution in the NEPHSP as long-term nationwide work led by governments
D Adaptability	2. Annual regional-tailored guidance plan had a good adaptability for falls prevention among older people		
E Trialability			2. Very few small-scale tests prior to the formal implementation but complemented by the experience learned from other service items in NEPHSP
F Complexity	3. Service providers had a clear recognition of the complexity of falls prevention for older people		
G Design Quality and Packaging		2. No performance assessment indicator<br>3. Poor integration within the same item and across other items	
H Cost	4. Financial support from central and local governments	4. Limited budget allocated to falls prevention for older people	
**II. Outer setting**			
A Patient Needs & Resources	5. Service providers had a good awareness of increasingly potential needs of falls prevention among older people<br>6. Older people with the experience of fall-related injuries had a stronger awareness of falls prevention	5. Service providers had a poor understanding of specific needs of local older people about falls prevention<br>6. Falls were not perceived by service providers as an independent health issue<br>7. Older people had limited knowledge of falls prevention<br>8. Older people had a fatalistic view of falls as an inevitable accident in life<br>9. Less-educated older people had poor accessibility of easy-to-understand and	

© 2022 Ye P et al. *JAMA Network Open.*

		practice information on falls prevention<br>10. Older people had a weak willingness to accept fall-prevention interventions	
B Cosmopolitanism		11. Short-term partnerships with other government departments in a few projects<br>12. A lack of collaboration with civil society organizations, business and the private sector, and academic institutions	
C Peer Pressure			3. No competitive pressure from the long-term nationwide work led by governments
D External Policy & Incentives	7. National health policies required the scale-up of falls prevention for older people	13. An absence of a national policy as an action plan or guideline for falls prevention for older people<br>14. No external incentives	
**III. Inner setting**			
A Structural Characteristics	8. Mature structural characteristics of enforcement body		
B Networks & Communications	9. Secure networks and reliable communications within enforcement body		
C Culture	10. Enforcement bodies shared central elements of culture in valuing primary health care		
D Implementation Climate			
1 Tension for Change		15. Controversy over the burden of falls among local older people due to the lack of comprehensive epidemiology data	
2 Compatibility	11. Service providers placed a high value on the potential contribution of falls prevention for older people	16. A lack of broad integration with current workflows	
3 Relative Priority		17. Service providers had negative attitudes about the priority of falls prevention for older people compared to other health issues in NESPHSP	
4 Organizational Incentives & Rewards		18. Weak and intangible incentives within organizations	
5 Goals and Feedback		19. Poor alignment of the feedback with goals	
6 Learning Climate	N/A	N/A	N/A
E Readiness for Implementation			
1 Leadership Engagement		20. Inactive leadership engagement	

© 2022 Ye P et al. *JAMA Network Open.*

2 Available Resources		21. A paucity of training and capacity building resources	
3 Access to knowledge and information		22. Service providers had challenges to acquiring theoretical concepts and professional skills involved in interventions and applying them to prevent falls for older people	
**IV. Characteristics of individuals**			
A Knowledge & Beliefs about the Intervention		23. Service providers had insufficient professional knowledge and skills of the intervention	
B Self-efficacy		24. Service providers with less working experience had a low self-efficacy	
C Individual Stage of Change	N/A	N/A	N/A
D Individual Identification with Organization	12. Service providers had a good sense of identity within their organizations		
E Other Personal Attributes			4. Not many opportunities for local staff to reflect on their personal attributes in the implementation course
**V. Process**			
A Planning	13. Plan was developed in advance one to three months		
B Engaging			
1 Opinion Leaders	14. Experienced senior staff or direct leaders were opinion leaders within organizations<br>15. Community opinion leaders had a strong influence on falls prevention for older people		
2 Formally appointed internal implementation leaders	16. Experienced senior staff or direct leaders were internal implementation leaders		
3 Champions			5. Indifference or resistance was overcome by the collective discussion and decision instead of individual champions
4 External Change Agents		25. Fewer opportunities for external change agents to formally influence or facilitate intervention decisions in a desirable direction	

© 2022 Ye P et al. *JAMA Network Open.*

C Executing			6. The deviation from the plan was usually very minimum based on the qualitative performance assessment
D Reflecting & Evaluating		26. Few quantitative feedback and evaluation	

© 2022 Ye P et al. *JAMA Network Open.*
